# What is causing the rising incidence of esophageal adenocarcinoma in the West and will it also happen in the East?

**DOI:** 10.1007/s00535-019-01593-7

**Published:** 2019-06-06

**Authors:** Kenneth E. L. McColl

**Affiliations:** University of Glasgow/Gartnavel General Hospital, 1053 Great Western Road, Glasgow, G12 0YN UK

**Keywords:** Oesophageal adenocarcinoma, Rising incidence, *H. pylori*, Central obesity

## Abstract

In the West, the incidence of esophageal adenocarcinoma, which is a long-term complication of damage by gastroesophageal reflux, has been rising over recent decades. Two main factors are likely to account for this increase. The first is the rising incidence of central obesity which promotes gastroesophageal reflux. The second is the falling incidence of *H. pylori* infection and associated atrophic gastritis which reduces the acidity and peptic activity of gastric juice, the main factors damaging to the esophageal mucosa. The rise in esophageal adenocarcinoma has been mirrored by a fall in gastric cancer consistent with *H. pylori* atrophic gastritis protecting from the former and predisposing to the latter. The incidence of gastric cancer in Japan is still above the level at which a rise in esophageal adenocarcinoma became apparent in the West. Esophageal adenocarcinoma is likely to rise in Japan also as the incidence of gastric cancer falls but the degree of rise will depend on a variety of other environmental and genetic factors.

## Introduction

There has been a marked and steady increase in the incidence of oesophageal adenocarcinoma in the Western world [1, 2]. This has been apparent, particularly in Western Europe and North America. The average annual increase in the cancer has ranged from 3.5% in Scotland to 8.1% in Hawaii. In the Western world, there has also been a simultaneous fall in the incidence of gastric adenocarcinoma, though that decline started many decades before the rise in the incidence of oesophageal adenocarcinoma became apparent [2]. Some of the recorded increase in incidence of oesophageal adenocarcinoma might be due to cancers at the gastro-oesophagael junction being more frequently classified as oesophageal rather than gastric cancers in recent years but this would only account for a small component of the marked changes seen.

To explore the possible reasons for the recent increase in incidence of oesophageal adenocarcinoma, it is necessary to understand the aetiology of this cancer. Oesophageal adenocarcinoma is believed to be a long-term complication of chronic damage to the oesophageal mucosa by reflux of gastric juice [3]. Unlike gastric columnar mucosa, the oesophageal squamous mucosa is unable to withstand the damaging effects of the acid and pepsin in gastric juice along with bile, which may also be present in the stomach. The gastric juice causes erosion and ulceration of the squamous mucosa and with ongoing damage, the squamous epithelium undergoes metaplasia to columnar type epithelium which is much less susceptible to the chemical insult of gastric juice. This metaplastic epithelium may resemble gastric cardia epithelium or that of the small and large bowel when it’s referred to as intestinal metaplasia. This metaplastic epithelium is referred to as Barrett’s oesophagus and has a propensity to develop dysplasia and progress to adenocarcinoma.

Accurate information regarding changes in incidence of gastro-oesophageal reflux disease is more difficult to obtain than that on esophageal cancer. The information available, however, does suggest that in the Western world, reflux disease has increased substantially over the last few decades and at a rate similar to that of oesophageal adenocarcinoma [4]. It seems likely, therefore, that the increase in incidence of oesophageal adenocarcinoma can be largely or entirely explained by a rising incidence in gastro-oesophageal reflux disease. We must now explore the possible explanations for the rising incidence of reflux disease in the Western world over recent decades.

Gastro-oesophageal reflux occurs when there is failure of the lower oesophageal sphincter mechanism to prevent gastric juice refluxing up into the oesophagus. One important factor in promoting such reflux is central obesity. There is a strong positive association between waist circumference or BMI and reflux symptoms [5, 6]. One important mechanism by which central obesity promotes gastro-oesophageal reflux is by increasing intra-abdominal pressure and the gastro-oesophageal pressure gradient and this causes an increase in the rate of flow of gastric juice into the oesophagus whenever the lower oesophageal sphincter is open [7]. This increased intra-abdominal pressure also impairs oesophageal clearance of gastric refluxate [7]. There has been a marked increase in incidence of central obesity in the Western world over the past few decades and this is likely to be one factor contributing to the increase in reflux disease, and thus that of oesophageal adenocarcinoma [4]. Obesity is also linked to an increasing incidence of other cancers and it is possible that obesity is promoting oesophageal carcinoma by humoral mechanisms in addition to its mechanical effects [8].

The degree to which the gastro-esophageal reflux will damage the oesophageal mucosa will depend upon the nature of the refluxate and, in particular, its concentration of acid, pepsin and bile. *Helicobacter pylori* infection can result in atrophic gastritis with a loss in parietal and chief cell numbers, and thus a reduction in the concentration of acid and pepsin secreted by the stomach. We have been interested in the possible role of *H. pylori *infection in the rising incidence of gastro-oesophageal reflux disease and oesophageal adenocarcinoma.

There is a strong negative association between *H. pylori* infection and gastroesophageal reflux disease documented in the Western world [9]. In addition, there is also a strong negative association between *Helicobacter pylori* infection and oesophageal adenocarcinoma with the incidence of the cancer in *H. pylori* positive subjects being only 30–50% of that in *H. pylori* negatives [10]. The prevalence of *H. pylori* infection in the Western world has been falling over recent decades and the rise in oesophageal adenocarcinoma might therefore be explained by the loss of a protective effect of the infection against reflux disease [11].

The most plausible mechanism by which *H. pylori* infection might prevent oesophageal damage by refluxing gastric juice is by reducing the concentrations of acid and pepsin in the juice as a consequence of the infection inducing atrophic gastritis. In order for the infection to produce a significant protective effect, it would need to produce this effect in the majority of *H. pylori* infected subjects. Many studies have reported the effects of *H. pylori* infection on the gastric mucosa and its secretory function but most of these studies have been performed on patients with specific diseases arising from the infection, such as duodenal or gastric ulcers or gastric cancer. There are very few studies which have examined the effect of the infection on gastric secretory function in the general population.

**Fig. 1:**
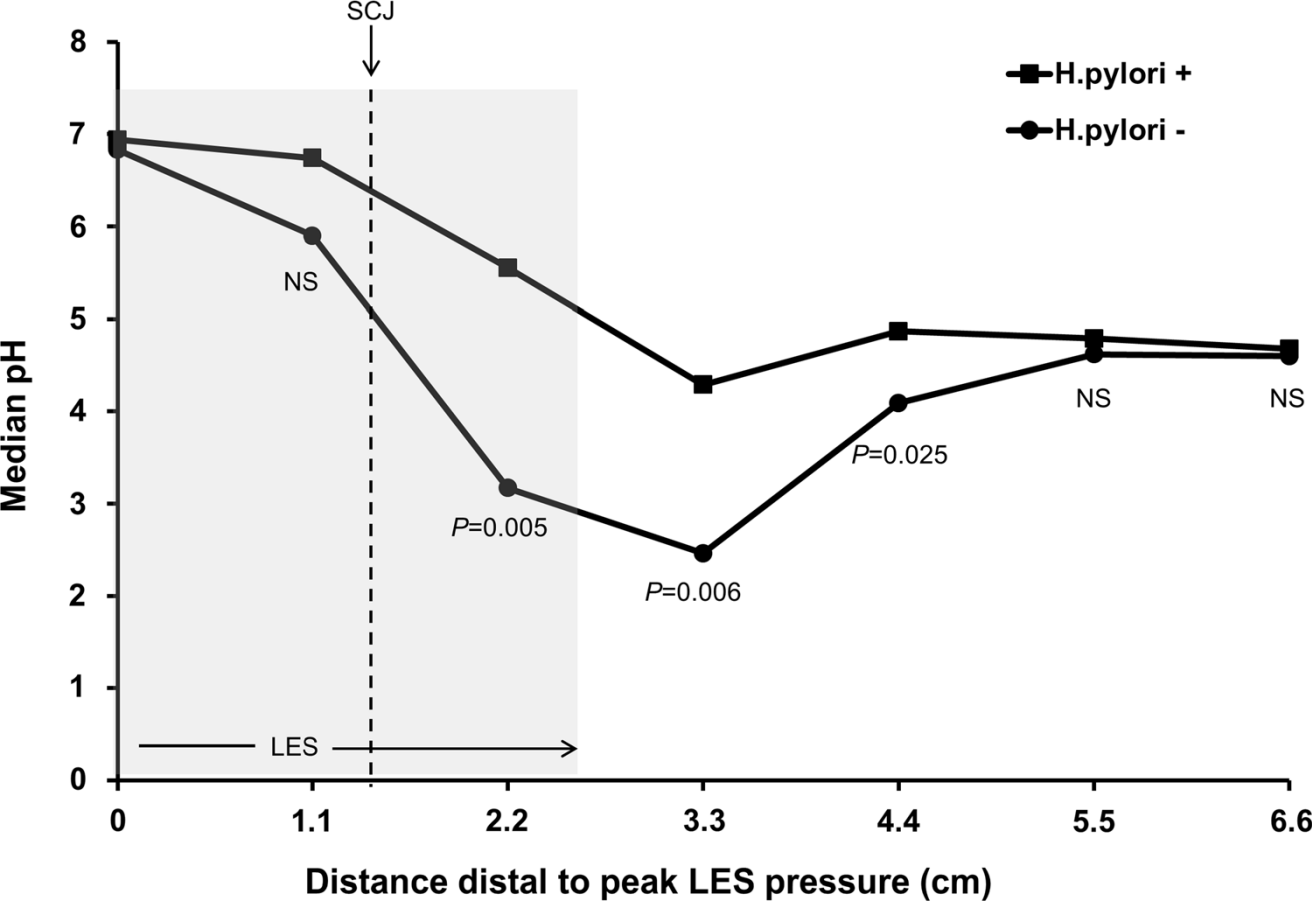
Median pH for 0–30 min period after meal relative to LES and SCJ in *H. pylori *positive (HP+) and negative (HP−) subjects. Reproduced by permission from Mitchell et al. [12]

We have recently investigated the association between *H. pylori i*nfection and gastric mucosal function in representative volunteers of our general populations in Scotland, which is a country which has experienced the changes in the incidence of oesophageal and gastric adenocarcinoma similar to the rest of the Western world. The studies were conducted in 28 *H. pylori* positive and 31 *H. pylori* negative subjects matched for age, sex and BMI [2]. The mean age of both groups was 55 years. We found that the density of parietal and chief cells from our infected volunteers was only approximately 50% of that in *H. pylori* negatives and that the reduction in cell density was most marked at the junction between the body and antral mucosa and also between the body and cardia mucosa proximally. We also studied intragastric acidity using high resolution pH-metry with 12 pH sensors cited throughout the stomach and this was undertaken both fasted and following a meal. In the *H. pylori* positive subjects, intragastric acidity was substantially less in all regions of the stomach compared to the *H. pylori* negatives under fasting conditions. Intragastric acidity was also substantially less in the *H. pylori* positives after the meal and this was most marked in the proximal stomach close to the gastroesophageal junction (Fig. 1). Under fasting conditions, the median pH in the *H. pylori* negative subjects close to the gastroesophageal junction was 2, compared to 6 in the *H. pylori* positives. Likewise, after the meal, the median intragastric pH close to the gastroesophageal junction in the *H. pylori* negatives remained at 2, whereas in the *H. pylori* positives it remained above 4. These studies demonstrate that the gastric juice of *H. pylori* infected subjects will be much less damaging to the oesophageal mucosa than that of uninfected subjects.

The above studies were performed in subjects with a mean age of 55 years and the gastric juice in younger subjects with *H. pylori* infection might have higher concentrations of acid and pepsin. It should, however, be remembered that incompetence of the lower oesophageal sphincter tends to occur with advancing years and, therefore, it will be the properties of the gastric juice at an age when reflux occurs that will be important.

The above population studies, therefore, provide strong evidence of a protective effect of *H. pylori* infection against the damaging effects of refluxing gastric juice and are consistent with the falling incidence of *H. pylori* infection and loss of this protective effect contributing to the rising incidence of reflux disease and its complication of oesophageal adenocarcinoma.

If the rising incidence of oesophageal adenocarcinoma is indeed due to a loss of the protective effects of *H. pylori* atrophic gastritis, then there should be a negative association between incidences of oesophageal adenocarcinoma and gastric adenocarcinoma for which *H. pylori* atrophic gastritis is the major risk factor. If *H. pylori* atrophic gastritis is both causing gastric cancer and protecting from oesophageal adenocarcinoma then we should see a negative association between the incidences of these two cancers. We have, therefore, recently investigated the association between oesophageal adenocarcinoma and gastric adenocarcinoma in collaboration with Forman et al. at the International Agency for Research into Cancer at Lyon [2].

**Fig. 2:**
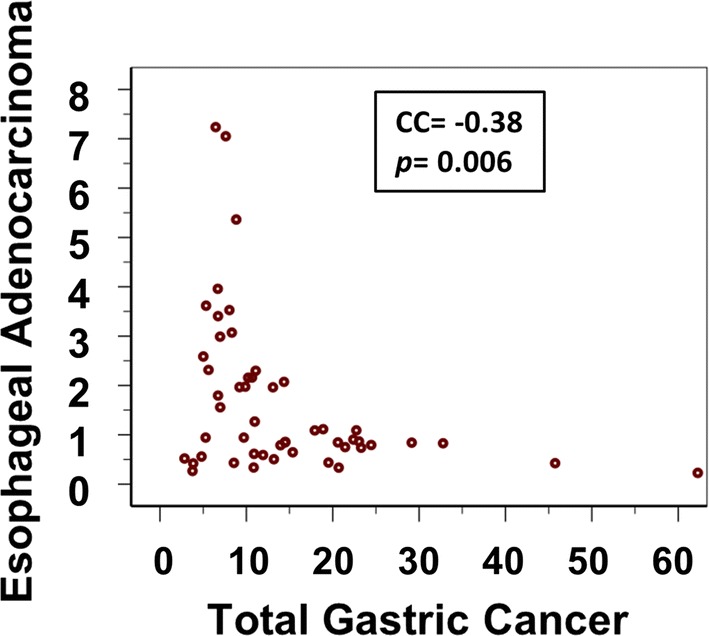
Correlations between incidence rates (WASR) of esophageal adenocarcinoma and gastric cancer in men. Each dot represents a dataset from an individual country. Reproduced by permission from Derakhshan et al. [2]

There is a large range in incidence of both oesophageal adenocarcinoma and gastric adenocarcinoma across different countries of the world. We have observed a strong negative correlation between the current incidence of gastric adenocarcinoma and oesophageal adenocarcinoma across these countries [2]. In countries with an incidence of gastric adenocarcinoma of more than 10, oesophageal adenocarcinoma was consistently low (Fig. 2). This is consistent with *H. pylori* atrophic gastritis predisposing to gastric cancer and protecting from esophageal adenocarcinoma. In countries with an incidence of gastric adenocarcinoma of less than 10, oesophageal adenocarcinoma was high but not always high. This is consistent with the absence of *H. pylori* induced atrophic gastritis being a permissive factor for esophageal adenocarcinoma but other promoting factors in particular gastroesophageal also being required.

We have also investigated the relationship between changing incidences of oesophageal adenocarcinoma and gastric adenocarcinoma over the past 20–50 years using populations from 38 different countries [2]. 66% of these countries showed a significant increase in gastric adenocarcinoma and 95% of the countries showed a significant decrease in gastric adenocarcinoma. In 28 of these 38 countries, there was a significant negative correlation between the changing incidence of the two cancers and no country showed a positive correlation.

These epidemiological studies therefore show a strong negative correlation between both current prevalences and changing incidences in oesophageal adenocarcinoma and gastric adenocarcinoma throughout the world [2]. These findings are consistent with *H. pylori* atrophic gastritis both predisposing to gastric adenocarcinoma and protecting from oesophageal adenocarcinoma and the falling incidence of *H. pylori* atrophic gastritis causing a falling incidence in gastric adenocarcinoma and a simultaneous rising incidence in gastric adenocarcinoma. These epidemiological studies combined with the physiological effects of *H. pylori* infection on gastric mucosal structure and function provide compelling evidence that the falling incidence of *H. pylori* infection is likely to be an important contributory factor to the rising incidence of oesophageal adenocarcinoma. The falling incidence of *H. pylori* infection along with the rise in the prevalence of central obesity are, therefore, two key factors explaining the recent increases in oesophageal adenocarcinoma observed in parts of the Western world.

The final and important question to be addressed is whether there will be a significant and substantial increase in oesophageal adenocarcinoma in Japan and other Eastern countries similar to that which has been seen in Western countries. As discussed above, the rise in oesophageal adenocarcinoma in Western countries and accompanying fall in gastric adenocarcinoma is thought to reflect the loss of protective effect of *H. pylori* atrophic gastritis. Japan currently still has a relatively high incidence of gastric adenocarcinoma and a very low incidence of oesophageal adenocarcinoma similar to the pattern seen in Western countries several decades ago. The incidence of gastric adenocarcinoma, however, is falling in Japan and will that herald an increase in oesophageal adenocarcinoma? Our epidemiological studies of the incidences of gastric and oesophageal adenocarcinoma indicated that a significant rise in oesophageal adenocarcinoma only appeared when the incidence of gastric adenocarcinoma fell below 10 [2]. The incidence of gastric adenocarcinoma in Japan remains above 10 and thus still above the level at which a rise in the incidence of oesophageal adenocarcinoma became apparent in the West. It is, therefore, too early to say whether the falling incidence of gastric adenocarcinoma will be accompanied by a significant rise in oesophageal adenocarcinoma in Japan.

Our epidemiological studies also indicated that a fall in gastric adenocarcinoma was not necessarily accompanied by a rise in oesophageal adenocarcinoma [2]. We interpret this as being consistent with a loss of atrophic gastritis and rise in acidity and peptic activity of gastric juice not necessarily being associated with a rise in oesophageal adenocarcinoma. This makes physiological sense as oesophageal damage depends not only on the acidity and peptic activity of the gastric juice but also upon failure of the lower oesophageal sphincter due to other factors such as central obesity. The extent to which the incidence of reflux disease and oesophageal adenocarcinoma are likely to rise in future decades in Japan following the disappearance of the protective effect of *H. pylori* infection and atrophic gastritis will depend upon the presence of other co-factors contributing to the pathogenesis of reflux disease such as central obesity. It should also be recognised that there are likely to be many other genetic and environmental factors other than *H. pylori* infection and central obesity contributing to the aetiology of reflux disease and its complication of oesophageal adenocarcinoma and the incidence of these in Japan will also determine the degree of increase in incidence of oesophageal adenocarcinoma in future decades.
